# Individual Differences in Alpha Frequency Drive Crossmodal Illusory Perception

**DOI:** 10.1016/j.cub.2014.11.034

**Published:** 2015-01-19

**Authors:** Roberto Cecere, Geraint Rees, Vincenzo Romei

**Affiliations:** 1 Centre for Brain Science, Department of Psychology, University of Essex, Wivenhoe Park, Colchester CO4 3SQ, UK; 2 Institute of Neuroscience and Psychology, University of Glasgow, 58 Hillhead Street, Glasgow G12 8QB, UK; 3 UCL Institute of Cognitive Neuroscience, University College London, 17 Queen Square, London WC1N 3AR, UK; 4 Wellcome Trust Centre for Neuroimaging at UCL, University College London, 12 Queen Square, London WC1N 3BG, UK

## Abstract

Perception routinely integrates inputs from different senses. Stimulus temporal proximity critically determines whether or not these inputs are bound together. Despite the temporal window of integration being a widely accepted notion, its neurophysiological substrate remains unclear. Many types of common audio-visual interactions occur within a time window of ∼100 ms [1–5]. For example, in the sound-induced double-flash illusion, when two beeps are presented within ∼100 ms together with one flash, a second illusory flash is often perceived [2]. Due to their intrinsic rhythmic nature, brain oscillations are one candidate mechanism for gating the temporal window of integration. Interestingly, occipital alpha band oscillations cycle on average every ∼100 ms, with peak frequencies ranging between 8 and 14 Hz (i.e., 120–60 ms cycle). Moreover, presenting a brief tone can phase-reset such oscillations in visual cortex [6, 7]. Based on these observations, we hypothesized that the duration of each alpha cycle might provide the temporal unit to bind audio-visual events. Here, we first recorded EEG while participants performed the sound-induced double-flash illusion task [4] and found positive correlation between individual alpha frequency (IAF) peak and the size of the temporal window of the illusion. Participants then performed the same task while receiving occipital transcranial alternating current stimulation (tACS), to modulate oscillatory activity [8] either at their IAF or at off-peak alpha frequencies (IAF±2 Hz). Compared to IAF tACS, IAF−2 Hz and IAF+2 Hz tACS, respectively, enlarged and shrunk the temporal window of illusion, suggesting that alpha oscillations might represent the temporal unit of visual processing that cyclically gates perception and the neurophysiological substrate promoting audio-visual interactions.

## Highlights

• Peak α frequency predicts temporal windows of the double-flash illusion
• tACS tuned around α frequency causally modulates this illusory temporal window
• Slower versus faster tACS α frequencies enlarged versus shrunk the illusory temporal window
• α peak is the “fingerprint” driving crossmodal impact upon visual processing

## Teaser

Multisensory integration occurs within a critical temporal window. Cecere et al. discover the neurophysiological correlates of this phenomenon. Individual oscillatory frequency within the occipital α band causally determines the individual temporal profile of cross-sensory integration, setting the sensory pace of conscious perceptual experience.

## Results and Discussion

### Individual Alpha Frequency Correlates with and Selectively Predicts the Temporal Profile of the Sound-Induced Double-Flash Illusion

**Figure 1:**
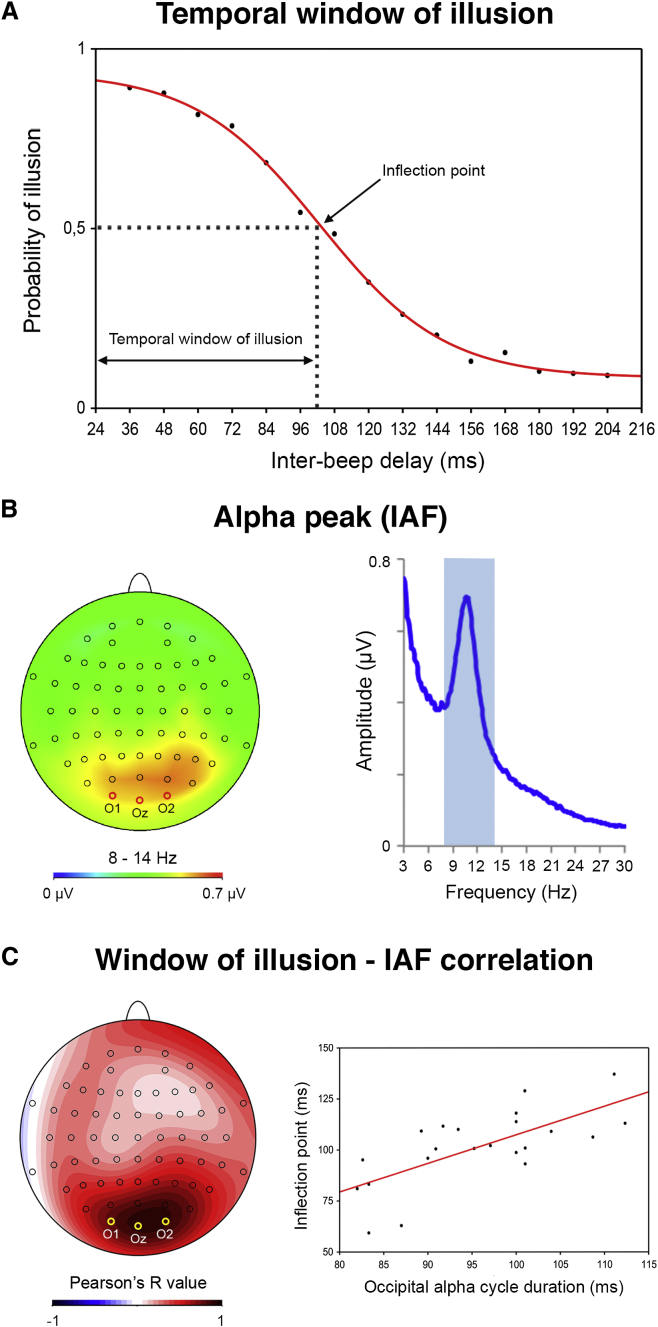
Individual Alpha Frequency Correlates with the Temporal Profile of the Double-Flash Illusion (A) Across-participants average probability of perceiving the illusion plotted as a function of interbeep delay. The red curve represents the sigmoid fit determining the amplitude of the window of illusion, corresponding to the inflection point of the sigmoid. (B) Across-participants average brain topography of oscillatory alpha activity during task performance and corresponding average FFT showing the peak frequency in the alpha band (light blue rectangle). (C) Scalp topography of the correlation index (Pearson’s *R*) between individual inflection points and alpha peak frequency (IAF) at each electrode, showing maximal correlation (r = 0.697; p < 0.001) around occipital electrodes (O1, O2, Oz). Scatterplot of the significant correlation between each individual’s inflection points (y axis) and the duration of one occipital alpha cycle (i.e., IAF; x axis).

To assess the correlation between individual alpha frequency (IAF) peak and the width of the temporal window of integration in which the illusion is perceived, we tested 22 healthy volunteers using a paradigm adapted from Shams et al. [4] where two beeps (7 ms duration) were played at different time delays between 36–204 ms (12 ms steps; see Supplemental Information and Figure S1 available online). A white disk was flashed for 12 ms below a fixation point time-aligned to the first beep, and participants reported whether they perceived one or two flashes. A sigmoid function was fitted to individual observations (see behavioral data analysis in Supplemental Information) to determine the inflection point of each participant’s behavioral curve, providing a reliable estimate of the temporal window in which the illusion was maximally perceived (average ∼100 ms; Figure 1A). EEG activity was recorded during the task and fast Fourier transform (FFT) used to calculate individual alpha frequency (IAF) peaks across the entire electrode array (Figure 1B). Inflection point values were then correlated with the width of IAF cycles revealing that these two measures were strongly and positively correlated with maxima over occipital electrodes (O1, O2, and Oz; n = 22, r = 0.697, regression slope = 1.4, y intercept = 0.34, p < 0.001; see Figure 1C), in line with our hypothesis.

It could be argued that the correlation found here is not selective for IAF peaks but results from general brain activity linked to the behavioral performance. If this would be the case, then the correlation we found may not necessarily serve as a conclusive explanation for our initial hypothesis, because IAF peak would not be the only parameter linked to the window of the illusion. According to this scenario, the positive correlation found here would also extend to other oscillatory parameters coregistered during the task. We therefore specifically tested to which extent the correlation of the illusory temporal profile was selective to the dimension hypothesized, i.e., alpha frequency peak. A first control analysis was performed within the alpha band and specifically looked at the correlation between the size of temporal window of the illusion and individual alpha power. In line with our initial hypothesis, this new control analysis confirmed the specificity of the effect for IAF peak, because the correlation with alpha power was not significant (r = 0.17, p = 0.45). When directly testing for any difference between our main correlation and the control correlation, we found them to be significantly different as expected (p < 0.04). We then extended our control analysis also to other frequency bands and specifically sought at any correlation between the size of the individual temporal window of the illusion and individual oscillatory frequency peaks in delta (1–3 Hz), theta (4–7 Hz), and beta (15–30 Hz) bands.

Again, we did not find any significant effect for any of these correlations (delta: r = 0.15, p = 0.50; theta: r = 0.06, p = 0.77; beta: r = −0.17, p = 0.44). Accordingly, this nonsignificant correlations were statistically different from the significant correlation between the temporal window of the illusion and IAF peak (all p values <0.03), confirming the specificity of our initial hypothesis, i.e., that individual alpha peak frequency is selectively linked to the temporal profile of the sound-induced double-flash illusion.

### Individual Alpha Peak Frequency Causally Shapes the Temporal Profile of the Sound-Induced Double-Flash Illusion

**Figure 2:**
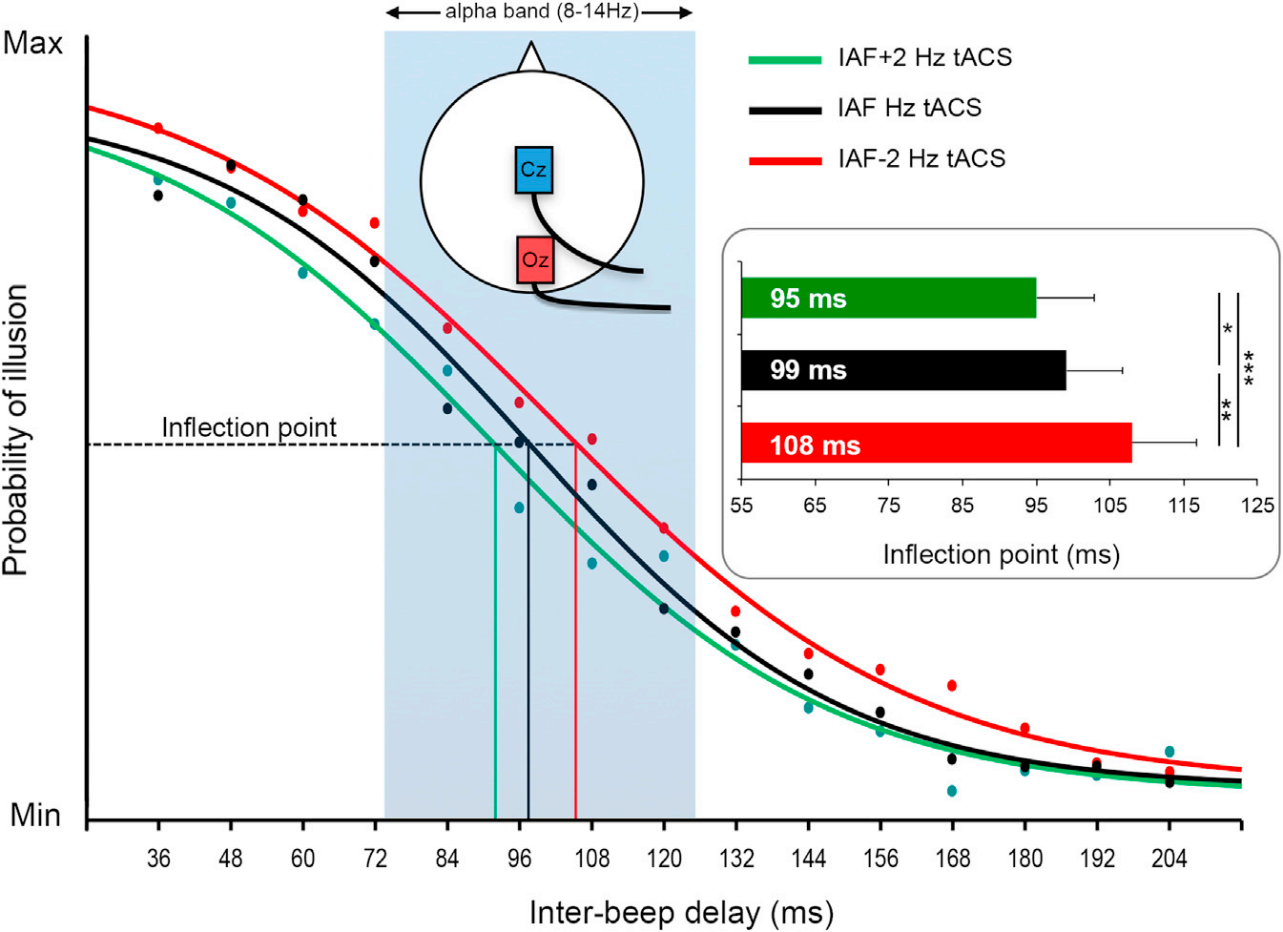
tACS at Different Frequencies Modulates the Size of the Temporal Window of Illusion The main plot shows the sigmoid fit (with aligned inflection points) of the average perceived illusion across participants (y axis) at different interbeep delays (x axis) in the three tACS conditions (Cz-Oz montage): tACS at IAF (black dots/curve), IAF+2 Hz (green dots/curve), and IAF−2 Hz (red dots/curve). Note that all the inflection points fall within the range of alpha frequency band, represented by the light-blue rectangle. Right inset shows the significant shifts of the average inflection points calculated for each participant sigmoid fit as a function of tACS condition. Error bars represent SEM. ^∗^p < 0.05; ^∗∗^p < 0.01; ^∗∗∗^p < 0.001.

In a second experiment, we sought causal evidence for a link between individual differences in IAF and the temporal window of the double-flash illusion. In 12 participants, we now delivered transcranial alternating current stimulation (tACS) over occipital cortex to modulate occipital oscillations [8] at their IAF or at slower (IAF−2 Hz) or faster (IAF+2 Hz) frequencies, i.e., far from IAF but still within the alpha band, while they were performing the flash-beep task (see Figure S2). If IAF causally determines the individual window of illusion (i.e., the inflection point of the sigmoid), then we hypothesized that driving IAF toward slower versus faster oscillations should result in wider versus shorter windows of illusion, respectively. Consistent with this hypothesis, repeated-measures ANOVA on inflection points (in ms) showed a main effect of tACS condition (F(2,22) = 10.11, p < 0.001, Figure 2). Post hoc paired t tests revealed that occipital tACS at IAF+2 Hz (92.7 ± 7.9 ms) significantly shrunk (t(11) = 1.82, p < 0.05, one-tailed), whereas IAF−2 Hz (106.4 ± 8.7 ms) significantly expanded (t(11) = 2.7, p = 0.01, one-tailed) the temporal window of the illusion relative to tACS at IAF (97.9 ± 7.6 ms) and relative to each other (t(11) = 4.29, p < 0.001, one-tailed). These tACS-dependent shifts in opposite directions suggest that IAF causally determines the temporal window of illusion.

**Figure 3:**
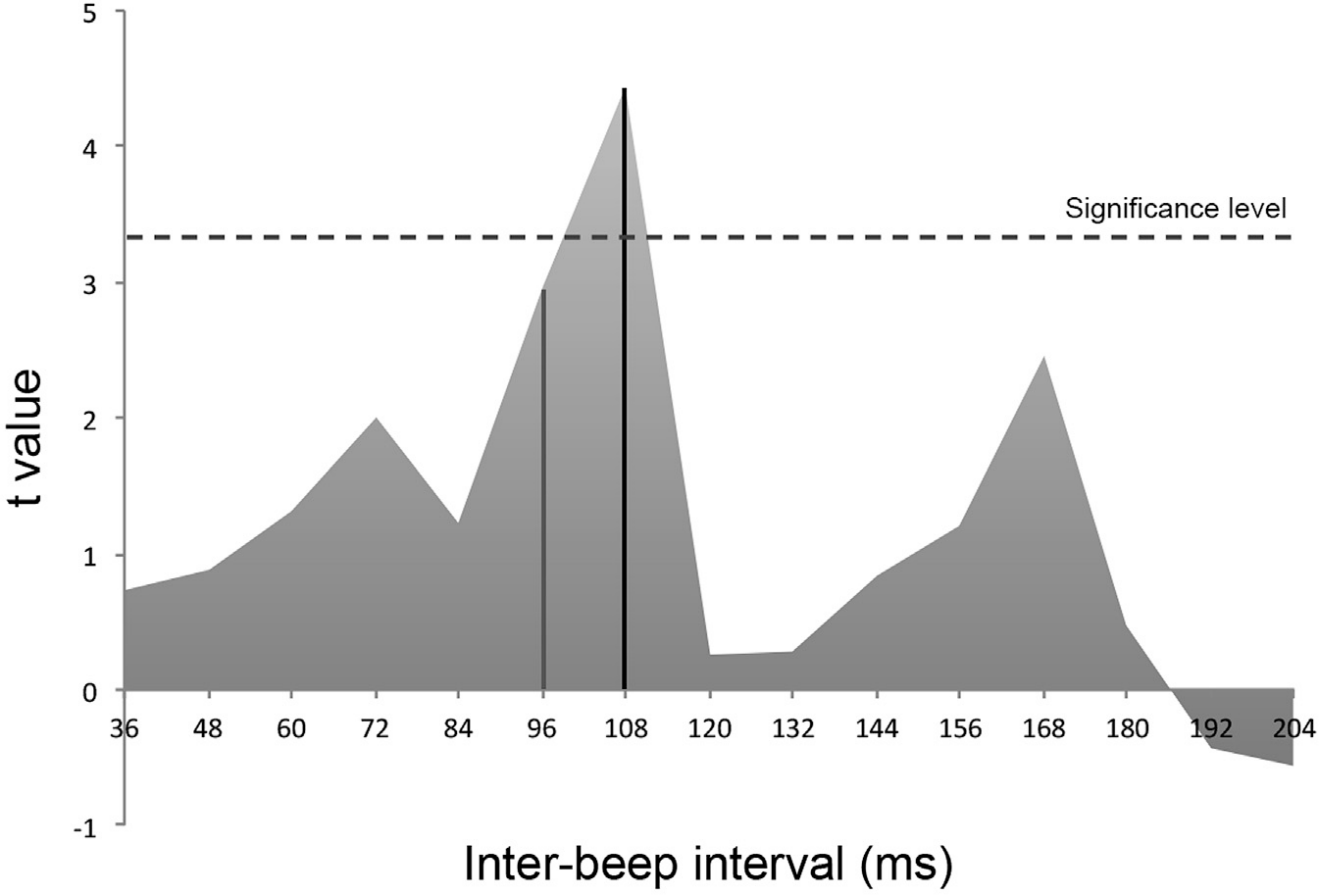
tACS at IAF±2 Selectively Modulates the Probability of Double-Flash Illusion around the Inflection Point, without Affecting the Overall Probability To assess whether tACS at different frequencies (IAF+2 versus IAF−2) induces a selective shift of inflection points versus an overall change in proneness to experience the double-flash illusion, we compared via t test the probability of illusion at each interbeep interval in the IAF±2 Hz tACS conditions. The graph shows that t values (y axis) were significantly different between the two tACS conditions only at 108 ms interbeep interval (t(11) = 4.4, p = 0.015), with a trend toward significance at 96 ms interbeep interval (t(11) = 2.98, p = 0.09). The probability of illusion at all the other interbeep intervals did not change between IAF±2 Hz conditions (all t values <2.4, all p values >0.23), demonstrating that tACS selectively shifts the inflection points but not the overall probability of experiencing the illusion.

It could be argued that present effects of tACS could be alternatively explained by shifts in the overall illusion susceptibility by tACS at IAF−2 Hz rather than our more specific windowing hypothesis. We reasoned that if these findings are the result of a general increase in the likelihood of the illusion at lower alpha frequency, then a differential probability of illusion between the ±2 Hz (i.e., the most extreme) conditions would be expected irrespective of the interbeep interval, i.e., not only around the inflection points but also at very short and very long stimulus onset asynchronies (SOAs). Alternatively, if the effect is determined by tACS windowing action, we would specifically predict a significant change in the probability of illusion only around the inflection points but not at the extreme interbeep intervals. We tested these two hypotheses using paired t tests to compare the probability of illusion between the ±2 Hz conditions at each interbeep interval. The results (see Figure 3) showed that the probability of illusion between ±2 Hz only differed at interbeep intervals around the inflection points (i.e., 100 ms). Specifically, 108 ms interbeep interval showed a significant difference between ±2 Hz (t(11) = 4.4, p = 0.015, one-tailed, Bonferroni corrected for 15 comparisons), whereas 96 ms interbeep interval showed a trend for a significant difference (t(11) = 2.98, p = 0.09, one-tailed, Bonferroni corrected). Crucially, the probability of illusion at all the other interbeep intervals did not change between ±2 Hz conditions (all t values <2.4, all p values >0.23, one-tailed, Bonferroni corrected).

Finally, to control whether any effect induced by tACS at the group level was genuinely reflected in systematic changes induced by the tACS manipulation at the individual level, we correlated each individual inflection point with the expected individually induced frequency of stimulation. As expected, we found significant positive correlations for tACS at IAF (n = 12, r = 0.71, regression slope = 1.75, y intercept = 0.28, p < 0.01), IAF+2 Hz (n = 12, r = 0.58, regression slope = 1.47, y intercept = 0.22, p < 0.05), and IAF−2 Hz (n = 12, r = 0.66, regression slope = 1.89, y intercept = 0.22, p < 0.02).

### A Multidimensional Oscillatory “Fingerprint” of the Human Visual System

The double-flash illusion has been linked to stronger activation of early visual areas [9, 10], as if a second real flash would have been presented [11]. Accordingly, acceleration of response times to the illusion is akin to that induced by physical flashes [12]. The illusory visual percept is mediated by early crossmodal interactions in low-level visual cortices [13], and its occurrence is predicted by alpha band occipital oscillatory amplitude [14, 15] enhanced coherence between auditory and visual areas [16] and is undistinguishable from real flashes in naive participants [17]. Moreover, proneness to this illusion has been linked to local gray matter volume in early visual cortices [18]. Finally, interventional approaches have identified that modulation of parieto-occipital areas interact with the illusion itself [19–21].

**Figure 4:**
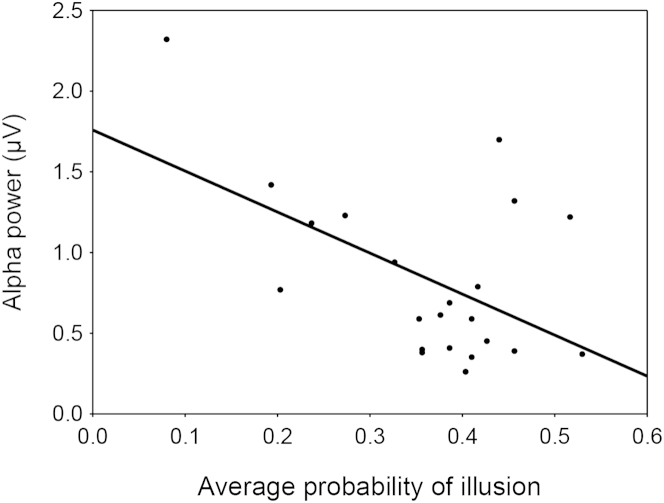
Alpha Power Is Inversely Correlated with Proneness to Experience the Sound-Induced Double-Flash Illusion Scatterplot of the correlation between average probability of perceiving the illusion across all SOAs (x axis) and individual alpha power at occipital electrodes (O1, O2, Oz; y axis).

If the occurrence of the illusion is predicted by the alpha oscillatory amplitude on a trial-by-trial basis [16], then the proneness to the illusion across participants in the present study may be similarly indexed by the individual amount of alpha power over occipital areas. Here, back to experiment 1, we differently analyzed our data and further tested this hypothesis by assessing the relationship between the proneness to the illusion (calculated as the overall probability of perceiving the illusion across the 15 temporal delays) and how this relates to another index of alpha oscillations, namely, its power. In line with the findings of Lange and colleagues [16], we found that higher levels of alpha power were inversely correlated with the proneness to perceive the illusion (r = 0.52, p < 0.015, see Figure 4). Intriguingly, in light of the existing literature these new findings predict that alpha power [16] and gray matter volume in early visual cortices [18] may be tightly linked, a hypothesis that needs direct empirical support. Moreover, this represents an important confirmation that adds to previous literature on the role of alpha power as a momentary index of cortical excitability [16, 22, 23] and alpha coherence between auditory and visual cortices as recently reported by Keil and colleagues [14].

Taken together, our results provide new evidence for early, low-level visual processing instantiating this illusion. We show how different indices of alpha oscillatory activity represent a multidimensional “fingerprint” of the human visual system and relate to different aspects of the perceived illusory flash. Specifically, we further confirm (and extend previous findings from a within-subjects design [16] to a between-subjects design) the role of alpha power as a momentary index of visual cortex excitability leading (low alpha) or not (high power) to (the illusory) perception. Importantly, we identify here for the first time the individual oscillatory “fingerprint” accounting for temporal windows of individual illusory perception. We provide evidence supporting the idea that occipital IAF is the neurophysiological biomarker that predicts and drives the temporal profile of the sound-induced double-flash illusion.

### A Mechanistic Account for Multisensory Interactions?

How does this biomarker engender the illusion? A visual stimulus is initially processed within a critical time window roughly corresponding to one alpha cycle [24], and a single-beep phase-resets occipital alpha activity [6, 7] by instantaneously enhancing visual cortex excitability [5, 6]. In the context of this illusion, a double beep phase-resets occipital alpha oscillations and enhances visual cortex excitability repeatedly. In other words, the instantaneous phase of the ongoing alpha will tend to be aligned to the consecutive sounds resulting in an increase in visual cortex excitability. When this crossmodal input happens at the same time as the presentation of a visual flash, it will interact with the ongoing visual processing by lowering the visual threshold and producing a reactivation/enhancement of the visual signal by sound, which is then erroneously interpreted by the brain as a new extra flash presented. Moreover, when more than two sounds are presented, sometimes even a third flash can be perceived, but seldom a fourth one [2]. In this respect, it might be the case that depending on the interbeep interval, a third auditory stimulus might still fall within an alpha cycle or may be tightly linked to it in a number of participants with slow individual alpha frequency, giving rise to a repeated illusory percept, an hypothesis that will require empirical support. Therefore, in this perspective, alpha oscillations represent the temporal unit of visual processing that could serve as a cortical scanning mechanism that cyclically gates perception through moments of inhibition and excitation [6, 25, 26]. In the specific case of the double-flash illusion, this scanning mechanism fails to provide accurate and veridical information as the timing of the sensory inputs is beyond its temporal resolution.

We speculate that this mechanism might extend to the touch-induced double-flash illusion [27], where the specific temporal influence of tactile stimulation may impact visual cortex excitability [28] and therefore visual processes as described above. More generally, such a mechanism could potentially explain a plethora of multisensory phenomena where the temporal information conveyed through visual stimuli is altered by concurrent presentation of auditory stimuli such as temporal ventriloquism [29, 30], simultaneity, and temporal and duration judgments (e.g., [18, 31]). In this respect, current interpretations of temporal processing and duration judgment, including the sound-induced double-flash illusion, have been generally discussed by postulating the existence of one or more internal clocks (e.g., [32–35]). However, recent findings in the field of visual perception have led to the development of rather modality-specific perspectives (e.g., [36–41]). But increasing evidence supports the notion that crossmodal stimulation might impact the activity of primary visual areas very early in time (e.g., [42–46]) and within a time frame strictly congruent with the present findings [5, 6]. Our findings provide evidence to support the notion of a modality-specific account. Specifically, we identified peak alpha frequency as the equivalent of an internal clock, possibly confined within the visual system and which is sensitive to crossmodal influences. Future research will shed light on whether this alpha clock times visual processing specifically or also generalizes to sensory processing in other modalities.

### Conclusions

Here, we provide a novel mechanistic account of how the striking sound-induced double-flash illusion is engendered. Specifically, using occipital oscillatory entrainment via tACS, we provide causal evidence that the temporal window of integration yielding the illusion is individually set by the frequency of occipital oscillations in the alpha band. In addition, we show that proneness to the illusion is linked to another dimension of alpha oscillations, namely, their power. Based on these findings, we suggest that the extra illusory flash is a by-product of the abrupt change in visual cortex excitability induced by the consecutive beeps within a critical temporal window for visual processing of the brief visual stimulus presented. This is likely the result of alpha phase alignments to the consecutive beeps while processing a visual stimulus, but taps onto a very similar mechanism as the one triggered by actual presentation of consecutive flashes, hence modifying our conscious experience.

## Supplemental Information

Document S1. Supplemental Discussion, Supplemental Experimental Procedures, and Figures S1 and S2

Document S2. Article plus Supplemental Information
