# Supplementary material for: Individual Differences in Alpha Frequency Drive Crossmodal Illusory Perception
Source: Curr Biol. 2015 Jan 19;25(2):231–5. doi: 10.1016/j.cub.2014.11.034 (PMC4300399; doi:10.1016/j.cub.2014.11.034)
Supplement: Document S1. Supplemental Discussion, Supplemental Experimental Procedures, and Figures S1 and S2 [file mmc1.pdf]

Current Biology, Volume *25*

Supplemental Information

Individual Differences in Alpha Frequency Drive Crossmodal Illusory Perception

Roberto Cecere, Geraint Rees, and Vincenzo Romei

Supplemental Information

Supplemental figures

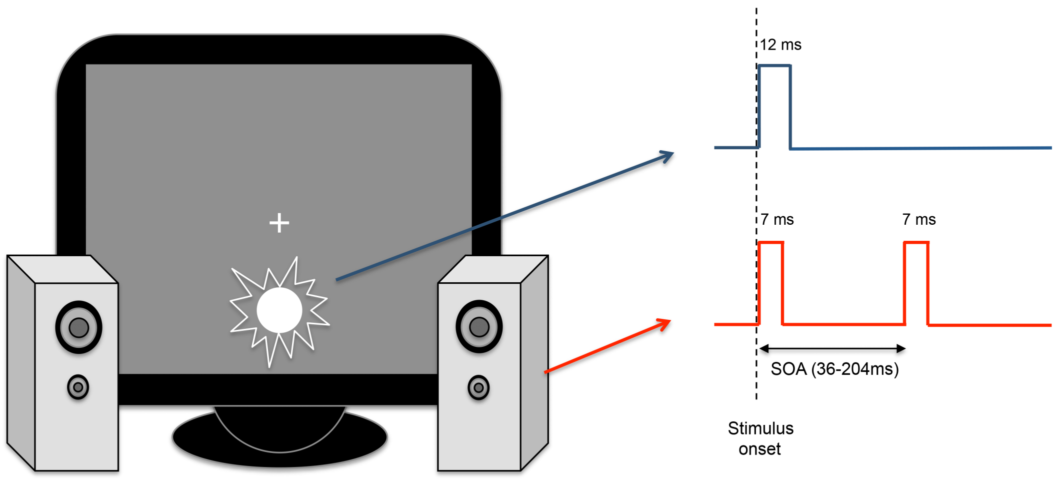

Figure S1, related to Figure 1. Schematic outline of experimental apparatus and paradigm. In each trial, participants were presented with one single visual flash accompanied by two beeps. The first beep was always temporally coincident with the visual flash, whereas the second beep could be presented at 15 different delays, ranging from 36 to 204ms in 12-ms steps. Participants reported whether they perceived one vs. two visual flashes.

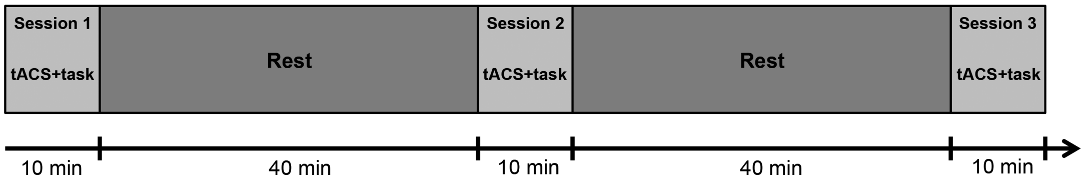

Figure S2, related to Figure 2. Schematic outline of the transcranial alternating current stimulation (tACS) protocol. In three separate sessions, participants received tACS over occipital areas at three different frequencies (IAF, IAF+2Hz, IAF-2Hz; counterbalanced order) for 10 min while performing the behavioral task. The tACS sessions were 40 min apart from each other.

Supplemental discussion

Occipital alpha phase-reset by sound sets the time window of the sound-induced double-flash illusion

According to our model, alpha phase reset by the first sound/visual stimulus would create a window of increased excitability lasting one alpha cycle and during which it is more likely to experience the illusory perception of a second flash. However, it is worth noting that neural oscillations are by definition rhythmic fluctuations between periods of maximal and minimal excitability, corresponding to the peaks and the troughs of the waves, and, more importantly, that the phase of alpha oscillations impacts perception (e.g. [S1]) and visual cortex excitability (e.g. [S2]). Hence, in our model, after the initial boost of excitability induced by the first sound/visual stimulus, a sinusoidal-like decay of the signal is expected over time, rather than a sharp drop. Behaviorally, this would in turn correspond to a reduced chance of perceiving a second flash as a function of time delay of the second sound within the alpha cycle.

Moreover, the behavioral performance we observed in the flash-beep task is the final result of many contributing factors that may introduce a certain amount of trial-by-trial variability. One example could be the phase reset mechanism we propose as a potential mechanism determining the window of the illusion. Other than a precise mechanism, it is rather a stochastic process where occipital phase reset is the most probable outcome of presenting a sound but not a mathematical

certainty. This can depend on a number of factors such as the phase of the ongoing oscillatory activity, i.e. it will be most difficult to phase reset an oscillatory activity that is 180 degrees out of phase at the time of the sound input. Other top-down components may in addition contribute to determine the slope of the sigmoid function depicted by the illusory percept at different inter-beep delays, e.g. a possible attention modulation or response bias around the edge of the perceivable illusion, due to the uncertainty of the illusory percept per se. In this case a response bias in one direction or another can be expected, depending on the criterion of each individual.

All in all, the above-mentioned factors will add a level of noise to our predicted phenomenon without overriding its fundamental characteristics. Therefore, although the model proposed here would predict a shift from probability of illusion = 1 to probability = 0 for inter-beep delay = 1 alpha cycle, this will follow a sigmoid (oscillatory) and not a quadratic function. Moreover, this model needs to also include additional levels of both bottom-up and top-down multisensory perceptual outcome that we empirically observed in our dataset.

The occipital vs. retinal nature of occipital tACS effects

The stimulation montage used in the current study is the same used by Kanai et al. [S3], demonstrating frequency specific effects of tACS stimulation on the likelihood of phosphene perception. These effects were later found to be due to stimulation of the retina [S4-6]. Following this observation, can the effects of our tACS manipulation alternatively result from retinal rather than cortical

stimulation? We can discard this possibility for a number of reasons outlined below.

In the first instance it is important to note that none of the participants reported experiencing phosphenes during the experimental procedure despite extensive debrief. Even in those few cases (3 out of 12) where phosphenes were reported, the tACS intensity was promptly lowered until no phosphene could be noticed further before the beginning of the task. Moreover, participants have been actively encouraged to report any flickering light sensation not only at the beginning of the experiment but also at any time during the experimental sessions. None of the participants (including those 3 participants for whom we lowered tACS intensity) reported having seen a phosphene throughout the end of the experimental sessions.

Since participants did not report awareness of any flash sensation during the stimulation, then any possible phosphene induction by the tACS would be below perceptual threshold by definition. However, given the subjective nature of phosphene reports, a "subthreshold phosphene" is not an invisible phosphene but the absence of a phosphene. As such, the absence of a subjective report is not expected to influence visual perception.

Second, we could assume that instead subthreshold stimulation might interfere with retinal function and this in turn might have created some indirect subthreshold sensory/retinal-induced cortical entrainment. But even if this were the case, this alternative explanation, which is very remote given the distance of the electrodes from the retina, would not weaken the interpretation of our

results. Instead it would result in an alternative explanation of the underlying mechanism.

Finally, it is worth noting that Neuling et al. [S7] (using the same montage of Kanai et al. [S3]) showed a clear localized effect of current density underneath the occipital electrode during tACS. This cannot be the result of any retinal-induced (but unperceived) effects of tACS. Moreover Helfrich et al. [S8] again using the same montage of Kanai et al. [S3] and our montage directly demonstrate cortical oscillatory entrainment during tACS at 10Hz. Again, this is not attributable to retinal induced phosphene perception.

Supplemental Experimental procedures

Participants

Thirty healthy volunteers were initially screened for their proneness and robustness of response to the sound-induced illusion. Eight participants were not included in the study because their performance could not be fitted to a sigmoid function (see below) due to very low proneness to or unreliable report of the illusion. The remaining twenty-two participants (mean age: 24.23, 15 females; 20 right-handed by self-report) had normal or corrected vision and normal hearing. Twelve participants (mean age: 26.33, 8 females) out of the twenty-two tested in the EEG experiment also took part in the tACS experiment (see below). All participants provided written informed consent and were paid to take part in the study, which was approved by the local ethics committee. Prior to participating in the tACS experiment, all subjects were screened for contraindications to tDCS/tACS using a self-report questionnaire.

Experimental setup

Stimuli were presented on a 17' CRT display (ViewSonic Graphics Series G90FB, refresh rate 85 Hz) in a dimly lit room. Participants sat in a comfortable chair in front of the monitor, at 57 cm viewing distance. Two small stereo PC speakers were placed on either side of the monitor and horizontally aligned with visual stimuli (Figure S1). Stimulus presentation and behavioral response recording were controlled by a PC running E-Prime software (version: 2; Psychology Software Tools, Pittsburgh, PA). Un-speeded manual two-choice responses were collected using a standard keyboard.

Stimuli and task

Stimuli and procedure were adapted from a previous study by Shams and colleagues [S9]. In all trials, both visual and auditory stimuli were presented. On each trial the visual stimulus consisted of a solid white circle subtending 2 degrees of visual angle. The auditory stimulus was a stereo, sinusoidal pure tone (frequency: 3.5 kHz; sampling rate: 44.1 kHz) of 7ms duration. Each trial started with display of a white fixation cross (0.7 visual degrees) centered on a uniform grey background. After a random time lag (500-1500ms), the visual stimulus was briefly flashed for 12ms, at 5 visual degrees eccentricity below fixation. On each trial, the single flash was always accompanied by two beeps: the first beep was always temporally aligned (i.e. synchronous) with the flash, whereas the second beep followed the first one with a random delay, chosen among 15 possible inter-beep delays (36, 48, 60, 72, 84, 96, 108, 120, 132, 144, 156, 168, 192, 204ms). The spatial configuration and the temporal profile of stimuli are illustrated in Figure S1. In a two-alternative forced choice paradigm, participants reported after each stimulus display whether they perceived one or two flashes by pressing the corresponding button on a keyboard (1 with index finger or 2 with middle finger, respectively). They were instructed to pay attention to visual stimuli only and to ignore the sounds, and to weigh accuracy over speed when responding. Each experimental block consisted of 300 trials (20 repetitions for each of the 15 inter-beep delays).

EEG experiment – paradigm and acquisition

In the EEG experiment participants performed one single block of the behavioral

task while continuous electroencephalography (EEG) was recorded from 64 sintered Ag/AgCl electrodes mounted on an elastic cap (EasyCap). The EEG signals were digitized at 2000 Hz and amplified using a Neuroscan SynAmps RT system (Compumedics, USA). Left mastoid was used as reference during acquisition.

tACS experiment – paradigm and brain stimulation

In the tACS experiment participants underwent three 10-min experimental sessions spaced 40 min apart from each other (Figure S2). In each session they performed one single block of the behavioral task while receiving continuous tACS at one of three possible frequencies, namely 1) the IAF as defined in the EEG experiment, 2) IAF-2Hz and 3) IAF+2Hz. The order of tACS sessions was counterbalanced across participants.

tACS was delivered by a battery-powered DC stimulator (Magstim, UK) through a pair of rubber electrodes enclosed in saline-soaked sponges and fixed on the head by elastic bands. The reference electrode was placed over the vertex (Cz in the international 10-20 EEG system), the stimulation electrode was placed over the occipital cortex (Oz). The reference electrode (Cz) had a larger size (35 cm^2) than the active electrode (Oz, 9 cm^2) to decrease current density delivered over Cz [S10]. The waveform of the current was sinusoidal, DC offset set at 0 and the intensity of the stimulation was set at 2 mA (10-sec fade in). The impedance was kept below 5 kΩ. All participants were actively encouraged to report any perception of tACS-induced phosphenes [S3, 4] throughout the experimental sessions. For participants reporting perception of phosphenes (N = 3), the

intensity was lowered in 0.1 mA steps until no phosphenes were perceived (mean stimulation intensity: 1.43 mA).

Behavioral data analysis

In both the EEG and tACS experiments, all responses from the behavioral task were used to calculate the temporal window in which the illusion was maximally perceived. To this end, the percentage of trials where the illusion (i.e. two flashes) was experienced was first plotted as a function of the inter-beep delay. A psychometric sigmoid function [$y = a+b/(1+\exp(-(x-c)/d))$; a = upper asymptote; b = lower asymptote; c = inflection point; d = slope] was then fitted to the data and the inter-beep delay (in ms) corresponding to the inflection point (centre) of the fitted sigmoid (i.e. the point of decay of the illusion) was considered as the amplitude of the window within which the illusion was experienced.

EEG data analysis

The EEG activity recorded during task execution was used to calculate the individual alpha peak frequency for each participant. The EEG data were down-sampled to 512 Hz and band-pass filtered (high pass filter: 3Hz; low pass filter: 40Hz). Continuous EEG signal was then segmented in artifact-free epochs of 1000ms (from -500ms to +500ms relative to the first stimulus onset in each trial) and re-referenced to the average of all electrodes. Based on previous reports showing perceptually relevant alpha modulation over posterior areas [S11-13], occipital electrodes (O1, O2 and Oz) were considered as region of interest (ROI) and pooled together prior to data analysis. For each participant and for all electrodes, including the ROI, a full power spectrum was obtained

through a Fast Fourier Transform (FFT) with zero-padded window (nominal frequency resolution 0.125Hz) and individual alpha frequency (IAF) was determined for each participant as the value corresponding to the maximum peak frequency within the 8-14 Hz range. Please note that in order to account for potential time lags between electrodes in the ROI the same analysis has been performed with the FFT analysis preceding pooling of electrodes. Results pointed to the same outcome, discounting any potential time lag issue.

Correlation analysis

Behavioral and electrophysiological data from the EEG experiment were used to explore whether IAF was predictive of the size of the temporal window of the double-flash illusion. Once IAF was identified using the procedure described above (see: EEG data analysis), we calculated for each participant the amplitude (in ms) of one single alpha cycle and performed a linear regression analysis between this measure and the individual width (in ms) of the temporal window of the illusion (see Behavioral data analysis).

tACS data analysis

Behavioral data from the tACS experiment were used to assess whether tACS at off-peak alpha frequencies (IAF±2Hz) significantly modulated the size of the temporal window of the illusion compared to tACS at IAF. A repeated-measures ANOVA was performed with tACS session (IAF, IAF-2Hz, IAF+2Hz) as a within-subject factor. The significant main effect of tACS session was examined using one-tailed paired *t*-tests with the assumption that tACS at IAF±2Hz modulated the size of the temporal window of the illusion in predicted directions, compared

to tACS at IAF. Following the same line of reasoning applied to the EEG experiment, to test whether changes in the temporal windows of the illusion genuinely reflected tACS-induced shifts within alpha frequency, three separate regression analyses were performed between the amplitude of the temporal window and the expected amplitude of one oscillatory cycle for each of the tACS conditions.

Supplemental references

S1. Busch, N.A., Dubois, J., and VanRullen, R. (2009). The phase of ongoing EEG oscillations predicts visual perception. J Neurosci *29*, 7869-7876.

S2. Romei, V., Gross, J., and Thut, G. (2012). Sounds reset rhythms of visual cortex and corresponding human visual perception. Curr Biol *22*, 807-813.

S3. Kanai, R., Chaieb, L., Antal, A., Walsh, V., and Paulus, W. (2008). Frequency-dependent electrical stimulation of the visual cortex. Curr Biol *18*, 1839-1843.

S4. Schwiedrzik, C.M. (2009). Retina or visual cortex? The site of phosphene induction by transcranial alternating current stimulation. Front Integr Neurosci *3*, 6.

S5. Schutter, D.J., and Hortensius, R. (2010). Retinal origin of phosphenes to transcranial alternating current stimulation. Clin Neurophysiol *121*, 1080-1084.

S6. Kar, K., and Krekelberg, B. (2012). Transcranial electrical stimulation over visual cortex evokes phosphenes with a retinal origin. J Neurophysiol *108*, 2173-2178.

S7. Neuling, T., Rach, S., and Herrmann, C.S. (2013). Orchestrating neuronal networks: sustained after-effects of transcranial alternating current stimulation depend upon brain states. Front Hum Neurosci *7*, 161.

S8. Helfrich, R.F., Schneider, T.R., Rach, S., Trautmann-Lengsfeld, S.A., Engel, A.K., and Herrmann, C.S. (2014). Entrainment of brain oscillations by transcranial alternating current stimulation. Curr Biol *24*, 333-339.

S9. Shams, L., Kamitani, Y., and Shimojo, S. (2002). Visual illusion induced by sound. Brain Res Cogn Brain Res *14*, 147-152.

S10. Nitsche, M.A., Cohen, L.G., Wassermann, E.M., Priori, A., Lang, N., Antal, A., Paulus, W., Hummel, F., Boggio, P.S., Fregni, F., et al. (2008). Transcranial direct current stimulation: State of the art 2008. Brain Stimul *1*, 206-223.

S11. Mathewson, K.E., Gratton, G., Fabiani, M., Beck, D.M., and Ro, T. (2009). To see or not to see: prestimulus alpha phase predicts visual awareness. J Neurosci *29*, 2725-2732.

S12. Romei, V., Brodbeck, V., Michel, C., Amedi, A., Pascual-Leone, A., and Thut, G. (2008). Spontaneous fluctuations in posterior alpha-band EEG activity reflect variability in excitability of human visual areas. Cereb Cortex *18*, 2010-2018.

S13. Romei, V., Rihs, T., Brodbeck, V., and Thut, G. (2008). Resting electroencephalogram alpha-power over posterior sites indexes baseline visual cortex excitability. Neuroreport *19*, 203-208.
