# Supplementary material for: Individual Differences in Alpha Frequency Drive Crossmodal Illusory Perception
Source: Curr Biol. 2015 Jan 19;25(2):231–5. doi: 10.1016/j.cub.2014.11.034 (PMC4300399; doi:10.1016/j.cub.2014.11.034)
Supplement: Document S2. Article plus Supplemental Information [file mmc2.pdf]

Current Biology 25, 231–235, January 19, 2015 ©2015 The Authors http://dx.doi.org/10.1016/j.cub.2014.11.034

Report

Individual Differences in Alpha Frequency Drive Crossmodal Illusory Perception

Roberto Cecere,[1,2] Geraint Rees,[3,4] and Vincenzo Romei[1,*]
[1]Centre for Brain Science, Department of Psychology, University of Essex, Wivenhoe Park, Colchester CO4 3SQ, UK
[2]Institute of Neuroscience and Psychology, University of Glasgow, 58 Hillhead Street, Glasgow G12 8QB, UK
[3]UCL Institute of Cognitive Neuroscience, University College London, 17 Queen Square, London WC1N 3AR, UK
[4]Wellcome Trust Centre for Neuroimaging at UCL, University College London, 12 Queen Square, London WC1N 3BG, UK

Summary

Perception routinely integrates inputs from different senses. Stimulus temporal proximity critically determines whether or not these inputs are bound together. Despite the temporal window of integration being a widely accepted notion, its neurophysiological substrate remains unclear. Many types of common audio-visual interactions occur within a time window of ~100 ms [1–5]. For example, in the sound-induced double-flash illusion, when two beeps are presented within ~100 ms together with one flash, a second illusory flash is often perceived [2]. Due to their intrinsic rhythmic nature, brain oscillations are one candidate mechanism for gating the temporal window of integration. Interestingly, occipital alpha band oscillations cycle on average every ~100 ms, with peak frequencies ranging between 8 and 14 Hz (i.e., 120–60 ms cycle). Moreover, presenting a brief tone can phase-reset such oscillations in visual cortex [6, 7]. Based on these observations, we hypothesized that the duration of each alpha cycle might provide the temporal unit to bind audio-visual events. Here, we first recorded EEG while participants performed the sound-induced double-flash illusion task [4] and found positive correlation between individual alpha frequency (IAF) peak and the size of the temporal window of the illusion. Participants then performed the same task while receiving occipital transcranial alternating current stimulation (tACS), to modulate oscillatory activity [8] either at their IAF or at off-peak alpha frequencies (IAF±2 Hz). Compared to IAF tACS, IAF−2 Hz and IAF+2 Hz tACS, respectively, enlarged and shrunk the temporal window of illusion, suggesting that alpha oscillations might represent the temporal unit of visual processing that cyclically gates perception and the neurophysiological substrate promoting audio-visual interactions.

Results and Discussion

Individual Alpha Frequency Correlates with and Selectively Predicts the Temporal Profile of the Sound-Induced Double-Flash Illusion

To assess the correlation between individual alpha frequency (IAF) peak and the width of the temporal window of integration in which the illusion is perceived, we tested 22 healthy volunteers using a paradigm adapted from Shams et al. [4] where two beeps (7 ms duration) were played at different time delays between 36–204 ms (12 ms steps; see Supplemental Information and Figure S1 available online). A white disk was flashed for 12 ms below a fixation point time-aligned to the first beep, and participants reported whether they perceived one or two flashes. A sigmoid function was fitted to individual observations (see behavioral data analysis in Supplemental Information) to determine the inflection point of each participant's behavioral curve, providing a reliable estimate of the temporal window in which the illusion was maximally perceived (average ~100 ms; Figure 1A). EEG activity was recorded during the task and fast Fourier transform (FFT) used to calculate individual alpha frequency (IAF) peaks across the entire electrode array (Figure 1B). Inflection point values were then correlated with the width of IAF cycles revealing that these two measures were strongly and positively correlated with maxima over occipital electrodes (O1, O2, and Oz; $n = 22$, $r = 0.697$, regression slope = 1.4, y intercept = 0.34, $p < 0.001$; see Figure 1C), in line with our hypothesis.

It could be argued that the correlation found here is not selective for IAF peaks but results from general brain activity linked to the behavioral performance. If this would be the case, then the correlation we found may not necessarily serve as a conclusive explanation for our initial hypothesis, because IAF peak would not be the only parameter linked to the window of the illusion. According to this scenario, the positive correlation found here would also extend to other oscillatory parameters coregistered during the task. We therefore specifically tested to which extent the correlation of the illusory temporal profile was selective to the dimension hypothesized, i.e., alpha frequency peak. A first control analysis was performed within the alpha band and specifically looked at the correlation between the size of temporal window of the illusion and individual alpha power. In line with our initial hypothesis, this new control analysis confirmed the specificity of the effect for IAF peak, because the correlation with alpha power was not significant ($r = 0.17$, $p = 0.45$). When directly testing for any difference between our main correlation and the control correlation, we found them to be significantly different as expected ($p < 0.04$). We then extended our control analysis also to other frequency bands and specifically sought at any correlation between the size of the individual temporal window of the illusion and individual oscillatory frequency peaks in delta (1–3 Hz), theta (4–7 Hz), and beta (15–30 Hz) bands.

Again, we did not find any significant effect for any of these correlations (delta: $r = 0.15$, $p = 0.50$; theta: $r = 0.06$, $p = 0.77$; beta: $r = -0.17$, $p = 0.44$). Accordingly, this nonsignificant correlations were statistically different from the significant correlation between the temporal window of the illusion and IAF peak (all p values <0.03), confirming the specificity of our initial hypothesis, i.e., that individual alpha peak frequency is selectively linked to the temporal profile of the sound-induced double-flash illusion.

Individual Alpha Peak Frequency Causally Shapes the Temporal Profile of the Sound-Induced Double-Flash Illusion

In a second experiment, we sought causal evidence for a link between individual differences in IAF and the temporal window

*Correspondence: vromei@essex.ac.uk
This is an open access article under the CC BY license (http://creativecommons.org/licenses/by/3.0/).

CrossMark

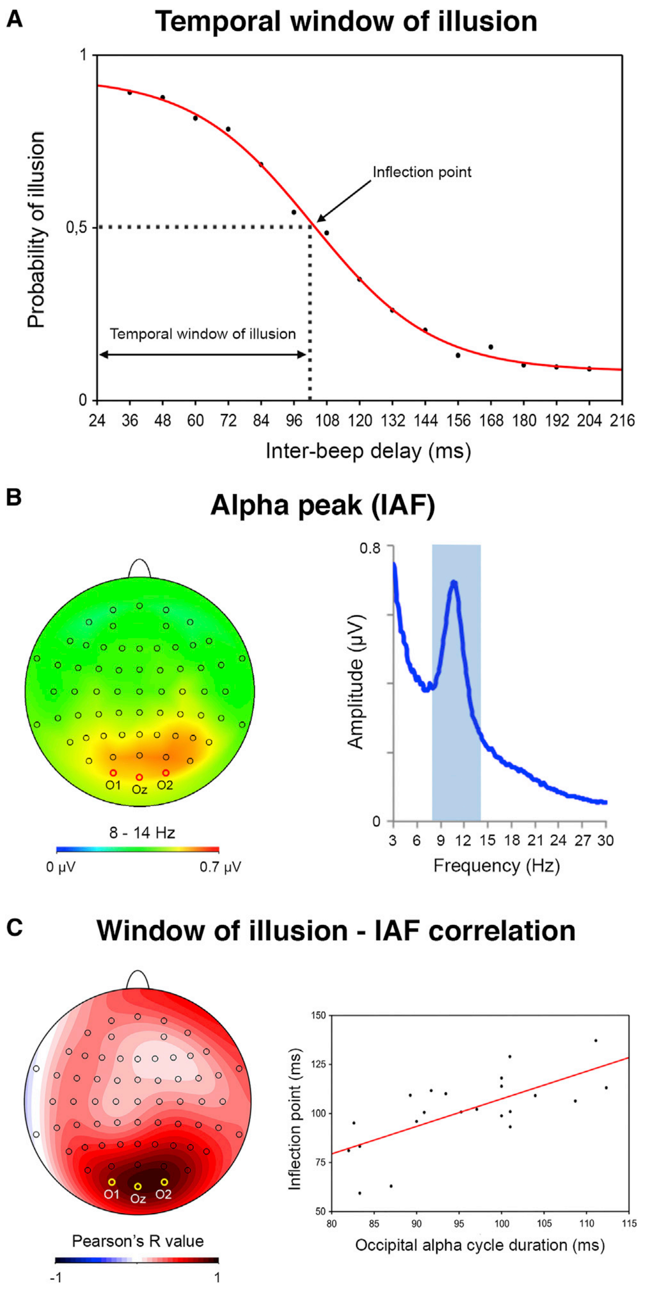

Figure 1. Individual Alpha Frequency Correlates with the Temporal Profile of the Double-Flash Illusion

(A) Across-participants average probability of perceiving the illusion plotted as a function of interbeep delay. The red curve represents the sigmoid fit determining the amplitude of the window of illusion, corresponding to the inflection point of the sigmoid.

(B) Across-participants average brain topography of oscillatory alpha activity during task performance and corresponding average FFT showing the peak frequency in the alpha band (light blue rectangle).

(C) Scalp topography of the correlation index (Pearson's *R*) between individual inflection points and alpha peak frequency (IAF) at each electrode, showing maximal correlation ($r = 0.697$; $p < 0.001$) around occipital electrodes (O1, O2, Oz). Scatterplot of the significant correlation between each individual's inflection points (y axis) and the duration of one occipital alpha cycle (i.e., IAF; x axis).

of the double-flash illusion. In 12 participants, we now delivered transcranial alternating current stimulation (tACS) over occipital cortex to modulate occipital oscillations [8] at their IAF or at slower (IAF−2 Hz) or faster (IAF+2 Hz) frequencies, i.e., far from IAF but still within the alpha band, while they were performing the flash-beep task (see Figure S2). If IAF causally determines the individual window of illusion (i.e., the inflection point of the sigmoid), then we hypothesized that driving IAF toward slower versus faster oscillations should result in wider versus shorter windows of illusion, respectively. Consistent with this hypothesis, repeated-measures ANOVA on inflection points (in ms) showed a main effect of tACS condition ($F(2,22) = 10.11$, $p < 0.001$, Figure 2). Post hoc paired t tests revealed that occipital tACS at IAF+2 Hz (92.7 ± 7.9 ms) significantly shrunk ($t(11) = 1.82$, $p < 0.05$, one-tailed), whereas IAF−2 Hz (106.4 ± 8.7 ms) significantly expanded ($t(11) = 2.7$, $p = 0.01$, one-tailed) the temporal window of the illusion relative to tACS at IAF (97.9 ± 7.6 ms) and relative to each other ($t(11) = 4.29$, $p < 0.001$, one-tailed). These tACS-dependent shifts in opposite directions suggest that IAF causally determines the temporal window of illusion.

It could be argued that present effects of tACS could be alternatively explained by shifts in the overall illusion susceptibility by tACS at IAF−2 Hz rather than our more specific windowing hypothesis. We reasoned that if these findings are the result of a general increase in the likelihood of the illusion at lower alpha frequency, then a differential probability of illusion between the ±2 Hz (i.e., the most extreme) conditions would be expected irrespective of the interbeep interval, i.e., not only around the inflection points but also at very short and very long stimulus onset asynchronies (SOAs). Alternatively, if the effect is determined by tACS windowing action, we would specifically predict a significant change in the probability of illusion only around the inflection points but not at the extreme interbeep intervals. We tested these two hypotheses using paired t tests to compare the probability of illusion between the ±2 Hz conditions at each interbeep interval. The results (see Figure 3) showed that the probability of illusion between ±2 Hz only differed at interbeep intervals around the inflection points (i.e., 100 ms). Specifically, 108 ms interbeep interval showed a significant difference between ±2 Hz ($t(11) = 4.4$, $p = 0.015$, one-tailed, Bonferroni corrected for 15 comparisons), whereas 96 ms interbeep interval showed a trend for a significant difference ($t(11) = 2.98$, $p = 0.09$, one-tailed, Bonferroni corrected). Crucially, the probability of illusion at all the other interbeep intervals did not change between ±2 Hz conditions (all t values <2.4, all p values >0.23, one-tailed, Bonferroni corrected).

Finally, to control whether any effect induced by tACS at the group level was genuinely reflected in systematic changes induced by the tACS manipulation at the individual level, we correlated each individual inflection point with the expected individually induced frequency of stimulation. As expected, we found significant positive correlations for tACS at IAF ($n = 12$, $r = 0.71$, regression slope = 1.75, y intercept = 0.28, $p < 0.01$), IAF+2 Hz ($n = 12$, $r = 0.58$, regression slope = 1.47, y intercept = 0.22, $p < 0.05$), and IAF−2 Hz ($n = 12$, $r = 0.66$, regression slope = 1.89, y intercept = 0.22, $p < 0.02$).

A Multidimensional Oscillatory "Fingerprint" of the Human Visual System

The double-flash illusion has been linked to stronger activation of early visual areas [9, 10], as if a second real flash would have been presented [11]. Accordingly, acceleration of response times to the illusion is akin to that induced by physical flashes [12]. The illusory visual percept is mediated by early crossmodal interactions in low-level visual cortices [13], and its

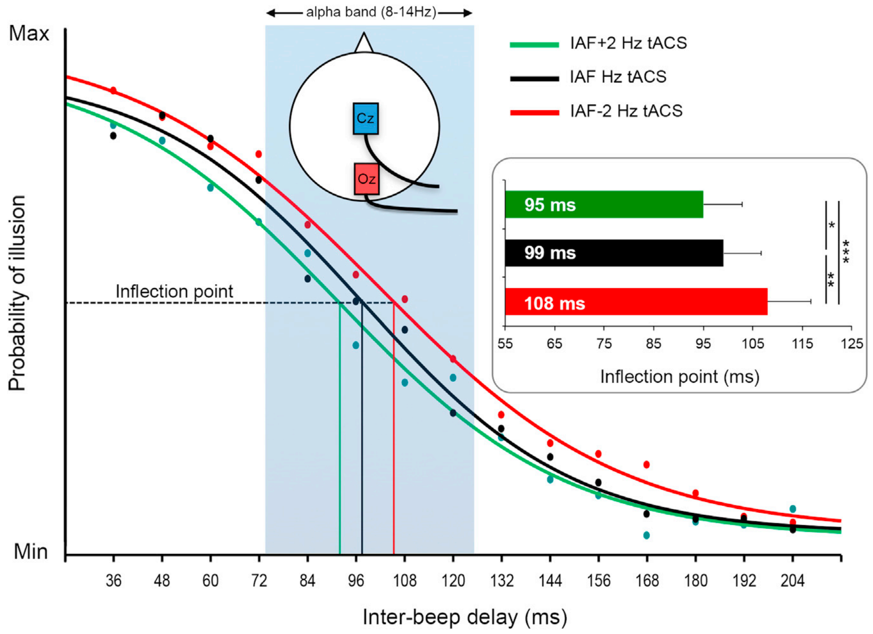

Figure 2. tACS at Different Frequencies Modulates the Size of the Temporal Window of Illusion

The main plot shows the sigmoid fit (with aligned inflection points) of the average perceived illusion across participants (y axis) at different interbeep delays (x axis) in the three tACS conditions (Cz-Oz montage): tACS at IAF (black dots/curve), IAF+2 Hz (green dots/curve), and IAF−2 Hz (red dots/curve). Note that all the inflection points fall within the range of alpha frequency band, represented by the light-blue rectangle. Right inset shows the significant shifts of the average inflection points calculated for each participant sigmoid fit as a function of tACS condition. Error bars represent SEM. *p < 0.05; **p < 0.01; ***p < 0.001.

occurrence is predicted by alpha band occipital oscillatory amplitude [14, 15] enhanced coherence between auditory and visual areas [16] and is undistinguishable from real flashes in naive participants [17]. Moreover, proneness to this illusion has been linked to local gray matter volume in early visual cortices [18]. Finally, interventional approaches have identified that modulation of parieto-occipital areas interact with the illusion itself [19–21].

If the occurrence of the illusion is predicted by the alpha oscillatory amplitude on a trial-by-trial basis [16], then the proneness to the illusion across participants in the present study may be similarly indexed by the individual amount of alpha power over occipital areas. Here, back to experiment 1, we differently analyzed our data and further tested this hypothesis by assessing the relationship between the proneness to the illusion (calculated as the overall probability of perceiving the illusion across the 15 temporal delays) and how this relates to another index of alpha oscillations, namely, its power. In line with the findings of Lange and colleagues [16], we found that higher levels of alpha power were inversely correlated with the proneness to perceive the illusion ($r = 0.52$, $p < 0.015$, see Figure 4). Intriguingly, in light of the existing literature these new findings predict that alpha power [16] and gray matter volume in early visual cortices [18] may be tightly linked, a hypothesis that needs direct empirical support. Moreover, this represents an important confirmation that adds to previous literature on the role of alpha power as a momentary index of cortical excitability [16, 22, 23] and alpha coherence between auditory and visual cortices as recently reported by Keil and colleagues [14].

Taken together, our results provide new evidence for early, low-level visual processing instantiating this illusion. We show how different indices of alpha oscillatory activity represent a multidimensional "fingerprint" of the human visual system and relate to different aspects of the perceived illusory

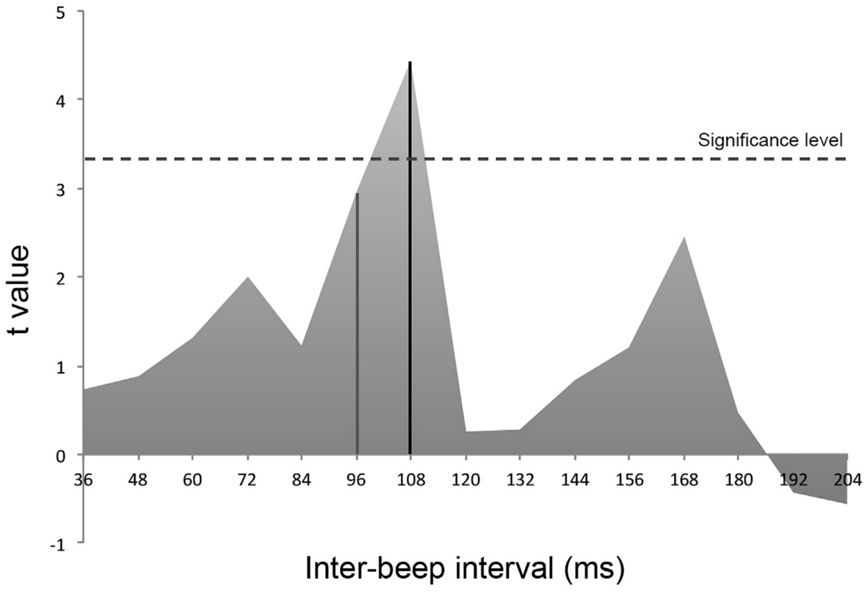

Figure 3. tACS at IAF±2 Selectively Modulates the Probability of Double-Flash Illusion around the Inflection Point, without Affecting the Overall Probability

To assess whether tACS at different frequencies (IAF+2 versus IAF−2) induces a selective shift of inflection points versus an overall change in proneness to experience the double-flash illusion, we compared via t test the probability of illusion at each interbeep interval in the IAF±2 Hz tACS conditions. The graph shows that t values (y axis) were significantly different between the two tACS conditions only at 108 ms interbeep interval ($t(11) = 4.4$, $p = 0.015$), with a trend toward significance at 96 ms interbeep interval ($t(11) = 2.98$, $p = 0.09$). The probability of illusion at all the other interbeep intervals did not change between IAF±2 Hz conditions (all t values <2.4, all p values >0.23), demonstrating that tACS selectively shifts the inflection points but not the overall probability of experiencing the illusion.

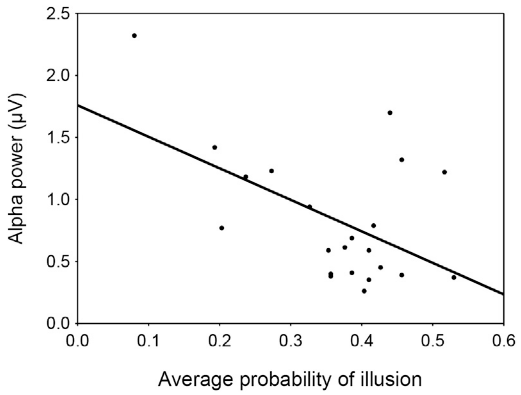

Figure 4. Alpha Power Is Inversely Correlated with Proneness to Experience the Sound-Induced Double-Flash Illusion

Scatterplot of the correlation between average probability of perceiving the illusion across all SOAs (x axis) and individual alpha power at occipital electrodes (O1, O2, Oz; y axis).

flash. Specifically, we further confirm (and extend previous findings from a within-subjects design [16] to a between-subjects design) the role of alpha power as a momentary index of visual cortex excitability leading (low alpha) or not (high power) to (the illusory) perception. Importantly, we identify here for the first time the individual oscillatory “fingerprint” accounting for temporal windows of individual illusory perception. We provide evidence supporting the idea that occipital IAF is the neurophysiological biomarker that predicts and drives the temporal profile of the sound-induced double-flash illusion.

A Mechanistic Account for Multisensory Interactions?

How does this biomarker engender the illusion? A visual stimulus is initially processed within a critical time window roughly corresponding to one alpha cycle [24], and a single-beep phase-resets occipital alpha activity [6, 7] by instantaneously enhancing visual cortex excitability [5, 6]. In the context of this illusion, a double beep phase-resets occipital alpha oscillations and enhances visual cortex excitability repeatedly. In other words, the instantaneous phase of the ongoing alpha will tend to be aligned to the consecutive sounds resulting in an increase in visual cortex excitability. When this crossmodal input happens at the same time as the presentation of a visual flash, it will interact with the ongoing visual processing by lowering the visual threshold and producing a reactivation/enhancement of the visual signal by sound, which is then erroneously interpreted by the brain as a new extra flash presented. Moreover, when more than two sounds are presented, sometimes even a third flash can be perceived, but seldom a fourth one [2]. In this respect, it might be the case that depending on the interbeep interval, a third auditory stimulus might still fall within an alpha cycle or may be tightly linked to it in a number of participants with slow individual alpha frequency, giving rise to a repeated illusory percept, an hypothesis that will require empirical support. Therefore, in this perspective, alpha oscillations represent the temporal unit of visual processing that could serve as a cortical scanning mechanism that cyclically gates perception through moments of inhibition and excitation [6, 25, 26]. In the specific case of the double-flash illusion, this scanning mechanism fails to provide accurate and veridical information as the timing of the sensory inputs is beyond its temporal resolution.

We speculate that this mechanism might extend to the touch-induced double-flash illusion [27], where the specific temporal influence of tactile stimulation may impact visual cortex excitability [28] and therefore visual processes as described above. More generally, such a mechanism could potentially explain a plethora of multisensory phenomena where the temporal information conveyed through visual stimuli is altered by concurrent presentation of auditory stimuli such as temporal ventriloquism [29, 30], simultaneity, and temporal and duration judgments (e.g., [18, 31]). In this respect, current interpretations of temporal processing and duration judgment, including the sound-induced double-flash illusion, have been generally discussed by postulating the existence of one or more internal clocks (e.g., [32–35]). However, recent findings in the field of visual perception have led to the development of rather modality-specific perspectives (e.g., [36–41]). But increasing evidence supports the notion that crossmodal stimulation might impact the activity of primary visual areas very early in time (e.g., [42–46]) and within a time frame strictly congruent with the present findings [5, 6]. Our findings provide evidence to support the notion of a modality-specific account. Specifically, we identified peak alpha frequency as the equivalent of an internal clock, possibly confined within the visual system and which is sensitive to crossmodal influences. Future research will shed light on whether this alpha clock times visual processing specifically or also generalizes to sensory processing in other modalities.

Conclusions

Here, we provide a novel mechanistic account of how the striking sound-induced double-flash illusion is engendered. Specifically, using occipital oscillatory entrainment via tACS, we provide causal evidence that the temporal window of integration yielding the illusion is individually set by the frequency of occipital oscillations in the alpha band. In addition, we show that proneness to the illusion is linked to another dimension of alpha oscillations, namely, their power. Based on these findings, we suggest that the extra illusory flash is a by-product of the abrupt change in visual cortex excitability induced by the consecutive beeps within a critical temporal window for visual processing of the brief visual stimulus presented. This is likely the result of alpha phase alignments to the consecutive beeps while processing a visual stimulus, but taps onto a very similar mechanism as the one triggered by actual presentation of consecutive flashes, hence modifying our conscious experience.

Supplemental Information

Supplemental Information includes Supplemental Discussion, Supplemental Experimental Procedures, and two figures and can be found with this article online at http://dx.doi.org/10.1016/j.cub.2014.11.034.

Acknowledgments

This work was supported by the Wellcome Trust (G.R.) and Essex Research Promotion Funds (V.R.). We would like to thank Roger Deeble for his invaluable technical support during the setup of the study.

Received: July 24, 2014
Revised: October 21, 2014
Accepted: November 13, 2014
Published: December 24, 2014

References

1. Meredith, M.A., Nemitz, J.W., and Stein, B.E. (1987). Determinants of multisensory integration in superior colliculus neurons. I. Temporal factors. J. Neurosci. *7*, 3215–3229.
2. Shams, L., Kamitani, Y., and Shimojo, S. (2000). Illusions. What you see is what you hear. Nature *408*, 788.
3. Slutsky, D.A., and Recanzone, G.H. (2001). Temporal and spatial dependency of the ventriloquism effect. Neuroreport *12*, 7–10.
4. Shams, L., Kamitani, Y., and Shimojo, S. (2002). Visual illusion induced by sound. Brain Res. Cogn. Brain Res. *14*, 147–152.
5. Romei, V., Murray, M.M., Merabet, L.B., and Thut, G. (2007). Occipital transcranial magnetic stimulation has opposing effects on visual and auditory stimulus detection: implications for multisensory interactions. J. Neurosci. *27*, 11465–11472.
6. Romei, V., Gross, J., and Thut, G. (2012). Sounds reset rhythms of visual cortex and corresponding human visual perception. Curr. Biol. *22*, 807–813.
7. Mercier, M.R., Foxe, J.J., Fiebelkorn, I.C., Butler, J.S., Schwartz, T.H., and Molholm, S. (2013). Auditory-driven phase reset in visual cortex: human electrocorticography reveals mechanisms of early multisensory integration. Neuroimage *79*, 19–29.
8. Helfrich, R.F., Schneider, T.R., Rach, S., Trautmann-Lengsfeld, S.A., Engel, A.K., and Herrmann, C.S. (2014). Entrainment of brain oscillations by transcranial alternating current stimulation. Curr. Biol. *24*, 333–339.
9. Watkins, S., Shams, L., Josephs, O., and Rees, G. (2007). Activity in human V1 follows multisensory perception. Neuroimage *37*, 572–578.
10. Watkins, S., Shams, L., Tanaka, S., Haynes, J.D., and Rees, G. (2006). Sound alters activity in human V1 in association with illusory visual perception. Neuroimage *31*, 1247–1256.
11. Shams, L., Kamitani, Y., Thompson, S., and Shimojo, S. (2001). Sound alters visual evoked potentials in humans. Neuroreport *12*, 3849–3852.
12. Fiedler, A., O'Sullivan, J.L., Schröter, H., Miller, J., and Ulrich, R. (2011). Illusory double flashes can speed up responses like physical ones: evidence from the sound-induced flash illusion. Exp. Brain Res. *214*, 113–119.
13. Mishra, J., Martinez, A., Sejnowski, T.J., and Hillyard, S.A. (2007). Early cross-modal interactions in auditory and visual cortex underlie a sound-induced visual illusion. J. Neurosci. *27*, 4120–4131.
14. Keil, J., Müller, N., Hartmann, T., and Weisz, N. (2014). Prestimulus beta power and phase synchrony influence the sound-induced flash illusion. Cereb. Cortex *24*, 1278–1288.
15. van Erp, J.B., Philippi, T.G., de Winkel, K.N., and Werkhoven, P. (2014). Pre- and post-stimulus EEG patterns associated with the touch-induced illusory flash. Neurosci. Lett. *562*, 79–84.
16. Lange, J., Keil, J., Schnitzler, A., van Dijk, H., and Weisz, N. (2014). The role of alpha oscillations for illusory perception. Behav. Brain Res. *271*, 294–301.
17. van Erp, J.B., Philippi, T.G., and Werkhoven, P. (2013). Observers can reliably identify illusory flashes in the illusory flash paradigm. Exp. Brain Res. *226*, 73–79.
18. de Haas, B., Cecere, R., Cullen, H., Driver, J., and Romei, V. (2013). The duration of a co-occurring sound modulates visual detection performance in humans. PLoS ONE *8*, e54789.
19. Bolognini, N., Rossetti, A., Casati, C., Mancini, F., and Vallar, G. (2011). Neuromodulation of multisensory perception: a tDCS study of the sound-induced flash illusion. Neuropsychologia *49*, 231–237.
20. Hamilton, R.H., Wiener, M., Drebing, D.E., and Coslett, H.B. (2013). Gone in a flash: manipulation of audiovisual temporal integration using transcranial magnetic stimulation. Front. Psychol. *4*, 571.
21. Kamke, M.R., Vieth, H.E., Cottrell, D., and Mattingley, J.B. (2012). Parietal disruption alters audiovisual binding in the sound-induced flash illusion. Neuroimage *62*, 1334–1341.
22. Romei, V., Brodbeck, V., Michel, C., Amedi, A., Pascual-Leone, A., and Thut, G. (2008). Spontaneous fluctuations in posterior alpha-band EEG activity reflect variability in excitability of human visual areas. Cereb. Cortex *18*, 2010–2018.
23. Romei, V., Rihs, T., Brodbeck, V., and Thut, G. (2008). Resting electroencephalogram alpha-power over posterior sites indexes baseline visual cortex excitability. Neuroreport *19*, 203–208.
24. Klimesch, W. (2011). Evoked alpha and early access to the knowledge system: the P1 inhibition timing hypothesis. Brain Res. *1408*, 52–71.
25. Mathewson, K.E., Gratton, G., Fabiani, M., Beck, D.M., and Ro, T. (2009). To see or not to see: prestimulus alpha phase predicts visual awareness. J. Neurosci. *29*, 2725–2732.
26. Klimesch, W., Sauseng, P., and Hanslmayr, S. (2007). EEG alpha oscillations: the inhibition-timing hypothesis. Brain Res. Brain Res. Rev. *53*, 63–88.
27. Violentyev, A., Shimojo, S., and Shams, L. (2005). Touch-induced visual illusion. Neuroreport *16*, 1107–1110.
28. Ramos-Estebanez, C., Merabet, L.B., Machii, K., Fregni, F., Thut, G., Wagner, T.A., Romei, V., Amedi, A., and Pascual-Leone, A. (2007). Visual phosphene perception modulated by subthreshold crossmodal sensory stimulation. J. Neurosci. *27*, 4178–4181.
29. Chen, L., and Vroomen, J. (2013). Intersensory binding across space and time: a tutorial review. Atten. Percept. Psychophys. *75*, 790–811.
30. Vroomen, J., de Gelder, B., and Vroomen, J. (2004). Temporal ventriloquism: sound modulates the flash-lag effect. J. Exp. Psychol. Hum. Percept. Perform. *30*, 513–518.
31. Romei, V., De Haas, B., Mok, R.M., and Driver, J. (2011). Auditory Stimulus Timing Influences Perceived duration of Co-Occurring Visual Stimuli. Front. Psychol. *2*, 215.
32. Ivry, R.B., and Richardson, T.C. (2002). Temporal control and coordination: the multiple timer model. Brain Cogn. *48*, 117–132.
33. Rosenbaum, D.A. (2002). Time, space, and short-term memory. Brain Cogn. *48*, 52–65.
34. Schöner, G. (2002). Timing, clocks, and dynamical systems. Brain Cogn. *48*, 31–51.
35. Wing, A.M. (2002). Voluntary timing and brain function: an information processing approach. Brain Cogn. *48*, 7–30.
36. Ghose, G.M., and Maunsell, J.H. (2002). Attentional modulation in visual cortex depends on task timing. Nature *419*, 616–620.
37. Johnston, A., Arnold, D.H., and Nishida, S. (2006). Spatially localized distortions of event time. Curr. Biol. *16*, 472–479.
38. Morrone, M.C., Ross, J., and Burr, D. (2005). Saccadic eye movements cause compression of time as well as space. Nat. Neurosci. *8*, 950–954.
39. Shuler, M.G., and Bear, M.F. (2006). Reward timing in the primary visual cortex. Science *311*, 1606–1609.
40. Yarrow, K., Haggard, P., Heal, R., Brown, P., and Rothwell, J.C. (2001). Illusory perceptions of space and time preserve cross-saccadic perceptual continuity. Nature *414*, 302–305.
41. Xuan, B., Zhang, D., He, S., and Chen, X. (2007). Larger stimuli are judged to last longer. J. Vis. *7*, 2.1–5.
42. Cappe, C., Thut, G., Romei, V., and Murray, M.M. (2010). Auditory-visual multisensory interactions in humans: timing, topography, directionality, and sources. J. Neurosci. *30*, 12572–12580.
43. Martuzzi, R., Murray, M.M., Michel, C.M., Thiran, J.P., Maeder, P.P., Clarke, S., and Meuli, R.A. (2007). Multisensory interactions within human primary cortices revealed by BOLD dynamics. Cereb. Cortex *17*, 1672–1679.
44. Noesselt, T., Tyll, S., Boehler, C.N., Budinger, E., Heinze, H.J., and Driver, J. (2010). Sound-induced enhancement of low-intensity vision: multisensory influences on human sensory-specific cortices and thalamic bodies relate to perceptual enhancement of visual detection sensitivity. J. Neurosci. *30*, 13609–13623.
45. Romei, V., Murray, M.M., Cappe, C., and Thut, G. (2009). Preperceptual and stimulus-selective enhancement of low-level human visual cortex excitability by sounds. Curr. Biol. *19*, 1799–1805.
46. Wang, Y., Celebrini, S., Trotter, Y., and Barone, P. (2008). Visuo-auditory interactions in the primary visual cortex of the behaving monkey: electrophysiological evidence. BMC Neurosci. *9*, 79.

Current Biology, Volume *25*

Supplemental Information

Individual Differences in Alpha Frequency Drive Crossmodal Illusory Perception

Roberto Cecere, Geraint Rees, and Vincenzo Romei

Supplemental Information

Supplemental figures

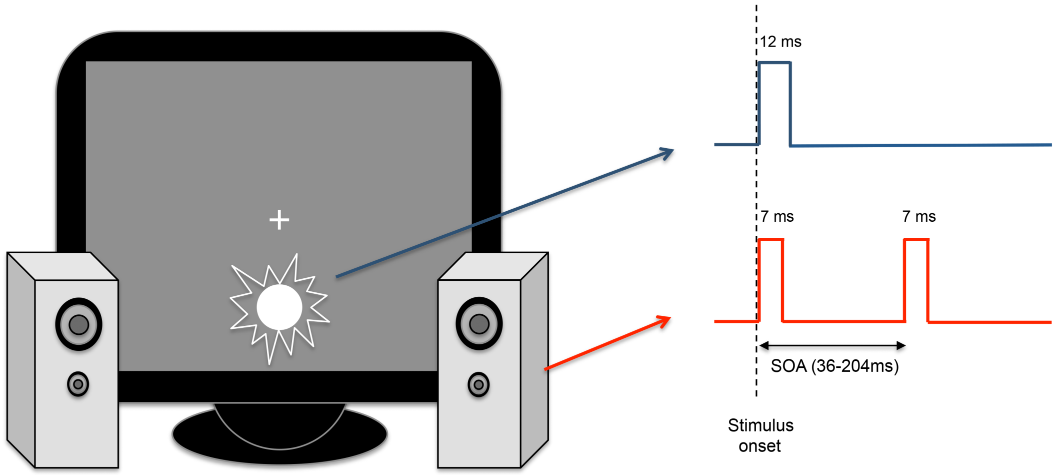

Figure S1, related to Figure 1. Schematic outline of experimental apparatus and paradigm. In each trial, participants were presented with one single visual flash accompanied by two beeps. The first beep was always temporally coincident with the visual flash, whereas the second beep could be presented at 15 different delays, ranging from 36 to 204ms in 12-ms steps. Participants reported whether they perceived one vs. two visual flashes.

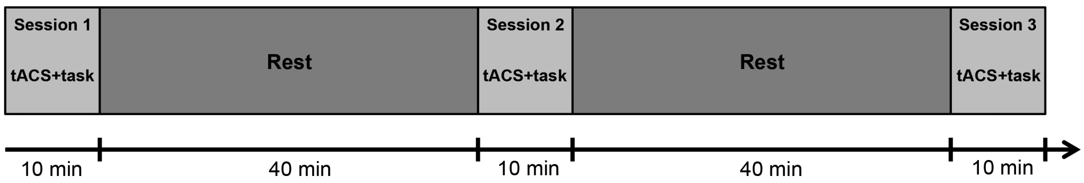

Figure S2, related to Figure 2. Schematic outline of the transcranial alternating current stimulation (tACS) protocol. In three separate sessions, participants received tACS over occipital areas at three different frequencies (IAF, IAF+2Hz, IAF-2Hz; counterbalanced order) for 10 min while performing the behavioral task. The tACS sessions were 40 min apart from each other.

Supplemental discussion

Occipital alpha phase-reset by sound sets the time window of the sound-induced double-flash illusion

According to our model, alpha phase reset by the first sound/visual stimulus would create a window of increased excitability lasting one alpha cycle and during which it is more likely to experience the illusory perception of a second flash. However, it is worth noting that neural oscillations are by definition rhythmic fluctuations between periods of maximal and minimal excitability, corresponding to the peaks and the troughs of the waves, and, more importantly, that the phase of alpha oscillations impacts perception (e.g. [S1]) and visual cortex excitability (e.g. [S2]). Hence, in our model, after the initial boost of excitability induced by the first sound/visual stimulus, a sinusoidal-like decay of the signal is expected over time, rather than a sharp drop. Behaviorally, this would in turn correspond to a reduced chance of perceiving a second flash as a function of time delay of the second sound within the alpha cycle.

Moreover, the behavioral performance we observed in the flash-beep task is the final result of many contributing factors that may introduce a certain amount of trial-by-trial variability. One example could be the phase reset mechanism we propose as a potential mechanism determining the window of the illusion. Other than a precise mechanism, it is rather a stochastic process where occipital phase reset is the most probable outcome of presenting a sound but not a mathematical

certainty. This can depend on a number of factors such as the phase of the ongoing oscillatory activity, i.e. it will be most difficult to phase reset an oscillatory activity that is 180 degrees out of phase at the time of the sound input. Other top-down components may in addition contribute to determine the slope of the sigmoid function depicted by the illusory percept at different inter-beep delays, e.g. a possible attention modulation or response bias around the edge of the perceivable illusion, due to the uncertainty of the illusory percept per se. In this case a response bias in one direction or another can be expected, depending on the criterion of each individual.

All in all, the above-mentioned factors will add a level of noise to our predicted phenomenon without overriding its fundamental characteristics. Therefore, although the model proposed here would predict a shift from probability of illusion = 1 to probability = 0 for inter-beep delay = 1 alpha cycle, this will follow a sigmoid (oscillatory) and not a quadratic function. Moreover, this model needs to also include additional levels of both bottom-up and top-down multisensory perceptual outcome that we empirically observed in our dataset.

The occipital vs. retinal nature of occipital tACS effects

The stimulation montage used in the current study is the same used by Kanai et al. [S3], demonstrating frequency specific effects of tACS stimulation on the likelihood of phosphene perception. These effects were later found to be due to stimulation of the retina [S4-6]. Following this observation, can the effects of our tACS manipulation alternatively result from retinal rather than cortical

stimulation? We can discard this possibility for a number of reasons outlined below.

In the first instance it is important to note that none of the participants reported experiencing phosphenes during the experimental procedure despite extensive debrief. Even in those few cases (3 out of 12) where phosphenes were reported, the tACS intensity was promptly lowered until no phosphene could be noticed further before the beginning of the task. Moreover, participants have been actively encouraged to report any flickering light sensation not only at the beginning of the experiment but also at any time during the experimental sessions. None of the participants (including those 3 participants for whom we lowered tACS intensity) reported having seen a phosphene throughout the end of the experimental sessions.

Since participants did not report awareness of any flash sensation during the stimulation, then any possible phosphene induction by the tACS would be below perceptual threshold by definition. However, given the subjective nature of phosphene reports, a “subthreshold phosphene” is not an invisible phosphene but the absence of a phosphene. As such, the absence of a subjective report is not expected to influence visual perception.

Second, we could assume that instead subthreshold stimulation might interfere with retinal function and this in turn might have created some indirect subthreshold sensory/retinal-induced cortical entrainment. But even if this were the case, this alternative explanation, which is very remote given the distance of the electrodes from the retina, would not weaken the interpretation of our

results. Instead it would result in an alternative explanation of the underlying mechanism.

Finally, it is worth noting that Neuling et al. [S7] (using the same montage of Kanai et al. [S3]) showed a clear localized effect of current density underneath the occipital electrode during tACS. This cannot be the result of any retinal-induced (but unperceived) effects of tACS. Moreover Helfrich et al. [S8] again using the same montage of Kanai et al. [S3] and our montage directly demonstrate cortical oscillatory entrainment during tACS at 10Hz. Again, this is not attributable to retinal induced phosphene perception.

Supplemental Experimental procedures

Participants

Thirty healthy volunteers were initially screened for their proneness and robustness of response to the sound-induced illusion. Eight participants were not included in the study because their performance could not be fitted to a sigmoid function (see below) due to very low proneness to or unreliable report of the illusion. The remaining twenty-two participants (mean age: 24.23, 15 females; 20 right-handed by self-report) had normal or corrected vision and normal hearing. Twelve participants (mean age: 26.33, 8 females) out of the twenty-two tested in the EEG experiment also took part in the tACS experiment (see below). All participants provided written informed consent and were paid to take part in the study, which was approved by the local ethics committee. Prior to participating in the tACS experiment, all subjects were screened for contraindications to tDCS/tACS using a self-report questionnaire.

Experimental setup

Stimuli were presented on a 17' CRT display (ViewSonic Graphics Series G90FB, refresh rate 85 Hz) in a dimly lit room. Participants sat in a comfortable chair in front of the monitor, at 57 cm viewing distance. Two small stereo PC speakers were placed on either side of the monitor and horizontally aligned with visual stimuli (Figure S1). Stimulus presentation and behavioral response recording were controlled by a PC running E-Prime software (version: 2; Psychology Software Tools, Pittsburgh, PA). Un-speeded manual two-choice responses were collected using a standard keyboard.

Stimuli and task

Stimuli and procedure were adapted from a previous study by Shams and colleagues [S9]. In all trials, both visual and auditory stimuli were presented. On each trial the visual stimulus consisted of a solid white circle subtending 2 degrees of visual angle. The auditory stimulus was a stereo, sinusoidal pure tone (frequency: 3.5 kHz; sampling rate: 44.1 kHz) of 7ms duration. Each trial started with display of a white fixation cross (0.7 visual degrees) centered on a uniform grey background. After a random time lag (500-1500ms), the visual stimulus was briefly flashed for 12ms, at 5 visual degrees eccentricity below fixation. On each trial, the single flash was always accompanied by two beeps: the first beep was always temporally aligned (i.e. synchronous) with the flash, whereas the second beep followed the first one with a random delay, chosen among 15 possible inter-beep delays (36, 48, 60, 72, 84, 96, 108, 120, 132, 144, 156, 168, 192, 204ms). The spatial configuration and the temporal profile of stimuli are illustrated in Figure S1. In a two-alternative forced choice paradigm, participants reported after each stimulus display whether they perceived one or two flashes by pressing the corresponding button on a keyboard (1 with index finger or 2 with middle finger, respectively). They were instructed to pay attention to visual stimuli only and to ignore the sounds, and to weigh accuracy over speed when responding. Each experimental block consisted of 300 trials (20 repetitions for each of the 15 inter-beep delays).

EEG experiment – paradigm and acquisition

In the EEG experiment participants performed one single block of the behavioral

task while continuous electroencephalography (EEG) was recorded from 64 sintered Ag/AgCl electrodes mounted on an elastic cap (EasyCap). The EEG signals were digitized at 2000 Hz and amplified using a Neuroscan SynAmps RT system (Compumedics, USA). Left mastoid was used as reference during acquisition.

tACS experiment – paradigm and brain stimulation

In the tACS experiment participants underwent three 10-min experimental sessions spaced 40 min apart from each other (Figure S2). In each session they performed one single block of the behavioral task while receiving continuous tACS at one of three possible frequencies, namely 1) the IAF as defined in the EEG experiment, 2) IAF-2Hz and 3) IAF+2Hz. The order of tACS sessions was counterbalanced across participants.

tACS was delivered by a battery-powered DC stimulator (Magstim, UK) through a pair of rubber electrodes enclosed in saline-soaked sponges and fixed on the head by elastic bands. The reference electrode was placed over the vertex (Cz in the international 10-20 EEG system), the stimulation electrode was placed over the occipital cortex (Oz). The reference electrode (Cz) had a larger size (35 cm^2) than the active electrode (Oz, 9 cm^2) to decrease current density delivered over Cz [S10]. The waveform of the current was sinusoidal, DC offset set at 0 and the intensity of the stimulation was set at 2 mA (10-sec fade in). The impedance was kept below 5 kΩ. All participants were actively encouraged to report any perception of tACS-induced phosphenes [S3, 4] throughout the experimental sessions. For participants reporting perception of phosphenes (N = 3), the

intensity was lowered in 0.1 mA steps until no phosphenes were perceived (mean stimulation intensity: 1.43 mA).

Behavioral data analysis

In both the EEG and tACS experiments, all responses from the behavioral task were used to calculate the temporal window in which the illusion was maximally perceived. To this end, the percentage of trials where the illusion (i.e. two flashes) was experienced was first plotted as a function of the inter-beep delay. A psychometric sigmoid function [$y = a+b/(1+\exp(-(x-c)/d))$; a = upper asymptote; b = lower asymptote; c = inflection point; d = slope] was then fitted to the data and the inter-beep delay (in ms) corresponding to the inflection point (centre) of the fitted sigmoid (i.e. the point of decay of the illusion) was considered as the amplitude of the window within which the illusion was experienced.

EEG data analysis

The EEG activity recorded during task execution was used to calculate the individual alpha peak frequency for each participant. The EEG data were down-sampled to 512 Hz and band-pass filtered (high pass filter: 3Hz; low pass filter: 40Hz). Continuous EEG signal was then segmented in artifact-free epochs of 1000ms (from -500ms to +500ms relative to the first stimulus onset in each trial) and re-referenced to the average of all electrodes. Based on previous reports showing perceptually relevant alpha modulation over posterior areas [S11-13], occipital electrodes (O1, O2 and Oz) were considered as region of interest (ROI) and pooled together prior to data analysis. For each participant and for all electrodes, including the ROI, a full power spectrum was obtained

through a Fast Fourier Transform (FFT) with zero-padded window (nominal frequency resolution 0.125Hz) and individual alpha frequency (IAF) was determined for each participant as the value corresponding to the maximum peak frequency within the 8-14 Hz range. Please note that in order to account for potential time lags between electrodes in the ROI the same analysis has been performed with the FFT analysis preceding pooling of electrodes. Results pointed to the same outcome, discounting any potential time lag issue.

Correlation analysis

Behavioral and electrophysiological data from the EEG experiment were used to explore whether IAF was predictive of the size of the temporal window of the double-flash illusion. Once IAF was identified using the procedure described above (see: EEG data analysis), we calculated for each participant the amplitude (in ms) of one single alpha cycle and performed a linear regression analysis between this measure and the individual width (in ms) of the temporal window of the illusion (see Behavioral data analysis).

tACS data analysis

Behavioral data from the tACS experiment were used to assess whether tACS at off-peak alpha frequencies (IAF±2Hz) significantly modulated the size of the temporal window of the illusion compared to tACS at IAF. A repeated-measures ANOVA was performed with tACS session (IAF, IAF-2Hz, IAF+2Hz) as a within-subject factor. The significant main effect of tACS session was examined using one-tailed paired *t*-tests with the assumption that tACS at IAF±2Hz modulated the size of the temporal window of the illusion in predicted directions, compared

to tACS at IAF. Following the same line of reasoning applied to the EEG experiment, to test whether changes in the temporal windows of the illusion genuinely reflected tACS-induced shifts within alpha frequency, three separate regression analyses were performed between the amplitude of the temporal window and the expected amplitude of one oscillatory cycle for each of the tACS conditions.

Supplemental references

S1. Busch, N.A., Dubois, J., and VanRullen, R. (2009). The phase of ongoing EEG oscillations predicts visual perception. J Neurosci *29*, 7869-7876.

S2. Romei, V., Gross, J., and Thut, G. (2012). Sounds reset rhythms of visual cortex and corresponding human visual perception. Curr Biol *22*, 807-813.

S3. Kanai, R., Chaieb, L., Antal, A., Walsh, V., and Paulus, W. (2008). Frequency-dependent electrical stimulation of the visual cortex. Curr Biol *18*, 1839-1843.

S4. Schwiedrzik, C.M. (2009). Retina or visual cortex? The site of phosphene induction by transcranial alternating current stimulation. Front Integr Neurosci *3*, 6.

S5. Schutter, D.J., and Hortensius, R. (2010). Retinal origin of phosphenes to transcranial alternating current stimulation. Clin Neurophysiol *121*, 1080-1084.

S6. Kar, K., and Krekelberg, B. (2012). Transcranial electrical stimulation over visual cortex evokes phosphenes with a retinal origin. J Neurophysiol *108*, 2173-2178.

S7. Neuling, T., Rach, S., and Herrmann, C.S. (2013). Orchestrating neuronal networks: sustained after-effects of transcranial alternating current stimulation depend upon brain states. Front Hum Neurosci *7*, 161.

S8. Helfrich, R.F., Schneider, T.R., Rach, S., Trautmann-Lengsfeld, S.A., Engel, A.K., and Herrmann, C.S. (2014). Entrainment of brain oscillations by transcranial alternating current stimulation. Curr Biol *24*, 333-339.

S9. Shams, L., Kamitani, Y., and Shimojo, S. (2002). Visual illusion induced by sound. Brain Res Cogn Brain Res *14*, 147-152.

S10. Nitsche, M.A., Cohen, L.G., Wassermann, E.M., Priori, A., Lang, N., Antal, A., Paulus, W., Hummel, F., Boggio, P.S., Fregni, F., et al. (2008). Transcranial direct current stimulation: State of the art 2008. Brain Stimul *1*, 206-223.

S11. Mathewson, K.E., Gratton, G., Fabiani, M., Beck, D.M., and Ro, T. (2009). To see or not to see: prestimulus alpha phase predicts visual awareness. J Neurosci *29*, 2725-2732.

S12. Romei, V., Brodbeck, V., Michel, C., Amedi, A., Pascual-Leone, A., and Thut, G. (2008). Spontaneous fluctuations in posterior alpha-band EEG activity reflect variability in excitability of human visual areas. Cereb Cortex *18*, 2010-2018.

S13. Romei, V., Rihs, T., Brodbeck, V., and Thut, G. (2008). Resting electroencephalogram alpha-power over posterior sites indexes baseline visual cortex excitability. Neuroreport *19*, 203-208.
